# Critical role of Lin28‐TNFR2 signalling in cardiac stem cell activation and differentiation

**DOI:** 10.1111/jcmm.14202

**Published:** 2019-02-07

**Authors:** Qiuling Xiang, Bicheng Yang, Li Li, Bin Qiu, Caihong Qiu, Xiao‐Bing Gao, Huanjiao (Jenny) Zhou, Wang Min

**Affiliations:** ^1^ Yale Stem Center Interdepartmental Program in Vascular Biology and Therapeutics Department of Pathology Yale University School of Medicine New Haven Connecticut; ^2^ Translational Medicine Center, the First Affiliated Hospital Sun Yat‐sen University Guangzhou Guangdong China; ^3^ Zhongshan School of Medicine Sun Yat‐sen University Guangzhou Guangdong China; ^4^ Center for Stem Cell Biology and Tissue Engineering Key Laboratory for Stem Cells and Tissue Engineering Ministry of Education, Sun Yat‐sen University Guangzhou Guangdong China; ^5^ Department of Comparative Medicine and Obstetrics, Gynecology, and Reproductive Sciences Yale University School of Medicine New Haven Connecticut

**Keywords:** cardiac stem cell, differentiation, Lin28, TNFR2

## Abstract

Tumour necrotic factor receptor‐2 (TNFR2) has been to be cardiac‐protective and is expressed in cardiac progenitor cells. Our goal is to define the mechanism for TNFR2‐mediated cardiac stem cell activation and differentiation. By employing a protocol of in vitro cardiac stem cell (CSC) differentiation from human inducible pluripotent stem cell (hiPSC), we show that expression of TNFR2 precedes expression of CSC markers followed by expression of mature cardiomyocyte proteins. Activation of TNFR2 by a specific agonist promotes whereas inhibition of TNFR2 by neutralizing antibody diminishes hiPSC‐based CSC differentiation. Interestingly, pluripotent cell factor RNA‐binding protein Lin28 enhances TNFR2 protein expression in early CSC activation by directly binding to a conserved Lin28‐motif within the 3'UTR of Tnfr2 mRNA. Furthermore, inhibition of Lin28 blunts TNFR2 expression and TNFR2‐dependent CSC activation and differentiation. Our study demonstrates a critical role of Lin28‐TNFR2 axis in CSC activation and survival, providing a novel strategy to enhance stem cell‐based therapy for the ischaemic heart diseases.

## 1 INTRODUCTION

Despite significant advances in treatment, coronary heart disease is the leading cause of morbidity and mortality worldwide, accounting for the deaths of 3.8 million men and 3.4 million women annually according to the World Health Organization^1^, ^2^. Although current therapeutic interventions for coronary heart disease improve clinical outcomes and prolong life, they are palliative in nature because they fail to address the fundamental issue of the loss of myocardium. In light of this, stem cell‐based therapies have gained increasing interest as a potential therapy for not only attenuating cardiac dysfunction but also affording myocardial regeneration^3^. Stem cell‐based therapy has applied to the treatment of myocardial infarcted in animal models and has generated promising results. It has been reported that stem cell‐based therapies could improve cardiac function, attenuated matrix remodelling, decrease infarct size and improve haemodynamic parameters in animal models and even in clinical trials. These two clinical trials have been reported^3^, ^4^, ^5^, ^6^. However, many hurdles have to be overcome before this strategy becomes practical. These hurdles include generating sufficient number of cardiac stem cell (CSC) and mature cardiomyocytes (CMs), and incorporating the cells efficiently and seamlessly into the host myocardium to ensure their synchronous contraction via electromechanical junctions. Therefore, a better understanding the regulation of stem cell‐derived differentiation of CSC/CMs is needed.

Based on currently available data and work from embryonic stem cells with in vivo lineage‐tracing results, a working model of heart cell lineage diversification has been recently proposed^7^. The BRY (Brachyury)^+^ mesoderm precursors differentiate early during development (embryonic day 3.25) into BRY^+^FLK1(foetal liver kinase 1)^+^ hemangioblasts and mesoderm posterior bHLH transcription factor‐1 (MESP1)^+^ primordial cardiovascular progenitor cells. After a second wave of FLK1 expression (E4.25), MESP^+^ cells develop into FLK1^+^ISL (islet‐1)^+^ multipotent cardiovascular progenitor cells^8^, ^9^ which can generate the three major types of cardiac cells: CMs, smooth muscle cells and endothelial cells^10^. CM commitment occurs with the induction of transcription factors such as NKX2.5 (NK2 transcription factor related, locus5) and GATA4 (GATA‐binding protein 4), which control its initial differentiation and further maturation^11^. A heart lineage map has been derived from relatively specific molecular markers, HCN4 (hyperpolarization‐activated cyclic nucleotide‐gated channel 4) for the first heart field which committed to cardiomyogenic cell lineage, ISL1 for second heart field which represent a multiple progenitors differentiating into various cell lineage in the heart, WT1 (wilms tumour 1) and TBX18 (T‐box family member 18) for the proepicardium, and WNT and PAX3 (paired box gene 3) for the neural crest^9^, ^10^, ^12^, ^13^, ^14^, ^15^, ^16^, ^17^, ^18^. Maturation of these CM precursor cells is characterized by the expression of cardiac contractile proteins such as myosin heavy chain (MHC) and cardiac troponin T (cTnT).

Tumour necrotic factor‐α (TNF) is a major mediator of inflammation and inflammatory diseases, and it has also been implicated in several cardiovascular diseases^19^. TNF elicits a broad spectrum of biological effects including proliferation, differentiation and apoptosis^20^, ^21^. These differences in TNF‐induced responses are mostly due to the differential signalling via its two distinct receptors: type I 55 kDa TNF receptor (TNFR1) and type II 75 kDa TNF receptor (TNFR2)^22^. TNFR1 is expressed ubiquitously, whereas TNFR2 expression is tightly regulated and found predominantly in CMs, vascular endothelial cells and haematopoietic cells^23^. Our in vitro and in vivo studies reveal that TNFR2 via Akt mediates cell survival and tissue repair^24^, ^25^. Our previous data have shown that in human ischaemic heart disease (IHD)TNFR2 and phospho‐histone H3 (pH3^S10^) dramatically increased. TNFR2^+^pH3^S10+^ CSCs are increased and co‐expressed pluripotent stem cell protein Lin28 in IHD, and these cells were CD45‐negative and VEGFR2‐negative. In vitro experiment showed hypoxia and/or TNF induce up‐regulation of TNFR2 and TNFR2^+^pH3^S10+^ CSCs^26^. These results suggest that both Lin28 and TNFR2 signalling may trigger CSC activation and differentiation. However, the functional connections between Lin28 and TNFR2 are not clear.

In the present study, we attempt to define the mechanism for TNFR2‐mediated CSC activation and differentiation. By employing a protocol of in vitro CSC differentiation from human inducible pluripotent stem cell (hiPSC), we show that TNFR2 is up‐regulated by pluripotent factor Lin28. Moreover, we demonstrate a critical role of Lin28‐TNFR2 axis in CSC activation and differentiation.

## 2 METHODS

### 2.1 Cardiomyocyte differentiation

To produce human CMs from pluripotent stem cells, hiPSCs were differentiated into hiPSC‐CMs with a chemically defined CM differentiation protocol^27^. Briefly, hiPSCs were first treated with a small molecule inhibitor of GSK3β signalling, CHIR99021 (STEMCELL Technologies Inc., Vancouver, Canada), to activate the Wnt signalling pathway. 2 days later, cells were treated with an inhibitor of Wnt signalling, IWP2 (R&D Systems, Minneapolis, MN, USA), until day 5. Afterward, RPMI/B‐27 medium without insulin (Life technologies Corporation, Carlsbad, CA, USA) was changed. From day 7 on, RPMI/B‐27 medium (Life technologies Corporation, Carlsbad, CA, USA) was changed every 2 days. Usually, robust spontaneous contraction occurred by day 12. Post‐differentiated cells should show hallmarks of CMs, including spontaneous contraction, cardiac‐specific gene and protein expression. The resulting CMs progressively matured over 30 days in culture based on myofilament expression pattern and mitotic activity. Functional maturity of the CMs were evaluated by electrophysiologic property of mature CMs through single cell dissection from random areas and followed by action potential and calcium influx recordings in the whole cell patchclamp configuration.

### 2.2 Quantitative RT‐PCR

Total RNA was prepared with RNeasy Plus Mini Kits and Qiashredder columns (Qiagen, Dusseldorf, Germany), as recommended by the manufacturer, and treated with DNase I (PromegaCorporation, Madison, CA, USA) for 15 min to eliminate potential contamination by genomic DNA. cDNA was generated by reverse transcription of total RNA (1000 ng) with iScript Advanced cDNA Synthesis Kits (Bio‐Rad, Berkeley, CA, USA). Quantitative RT‐PCR was performed and analysed by kinetic real‐time PCR with an ABI Prism 7900 system (Applied Biosystems). iQSYBR Green Supermix (Bio‐Rad, Berkeley, CA, USA) was used for relative quantification of the indicated genes. Expression data were normalized to the level of human GAPDH transcripts. The primers (NKX2.5, GATA4, SOX2, Nanog, OCT4, TNFR2, TNFR1, 18sRNA) for quantitative RT‐PCR are listed in Table S1.

### 2.3 Immunofluorescence‐staining analysis

Cells or frozen tissue slides were fixed with 4% paraformaldehyde, permeabilized with 0.2% Triton X‐100 in PBS, blocked with a solution of protein blocker for an hour and incubated with primary antibodies at 4°C overnight. Antibodies used are listed in Table S2. Secondary antibodies conjugated with Alexa Fluor 488 or 594 (Invitrogen, Carlsbad, USA) were then added, and the incubation was performed at room temperature for an hour in the dark. Nuclei were stained with 4'6‐diamidino‐2‐phenylindole (DAPI) (Vector Laboratories, Burlingame, CA, USA).

### 2.4 Immunoblotting and antibodies

Frozen tissues or cultured CMs after various treatments were lysed by sonication in 1.5 mL of cold lysis buffer (50 mmol/L Tris‐HCl, pH 7.6, 150 mmol/L NaCl, 0.1% Triton X‐100, 0.75% Brij 96, 1 mmol/L sodium orthovanadate, 1 mmol/L sodium fluoride, 1 mmol/L sodium pyrophosphate, 10 μg/mL aprotinin, 10 μg/mL leupeptin, 2 mmol/L PMSF, 1 mmol/L EDTA) and incubated on ice for 20 min. The cell lysates were subjected to SDS‐PAGE followed by immunoblotting (Immobilon P, Millipore, Milford, MA, USA). The chemiluminescence was detected using an ECL kit (Amersham Life Science, Arlington Heights, IL, USA). Antibodies used are listed in Table S2.

### 2.5 Statistical analysis

All figures are representative of at least three experiments unless otherwise noted. All graphs report mean ± SEM values of biological replicates. Comparisons between two groups were performed by unpaired, two‐tailed *t* test, between more than two groups by one‐way ANOVA followed by Bonferroni's post‐hoc or by two‐way ANOVA using Prism 6.0 software (GraphPad). *P* values were two‐tailed and values <0.05 were considered to indicate statistical significance. *P *<* *0.05, *P *<* *0.01 and *P *<* *0.001 are designated in all figures with *, **, ***, respectively.

## 3 RESULTS

### 3.1 Differentiation of hESCs and iPS cells into CSC and CMs

**Figure 1:**
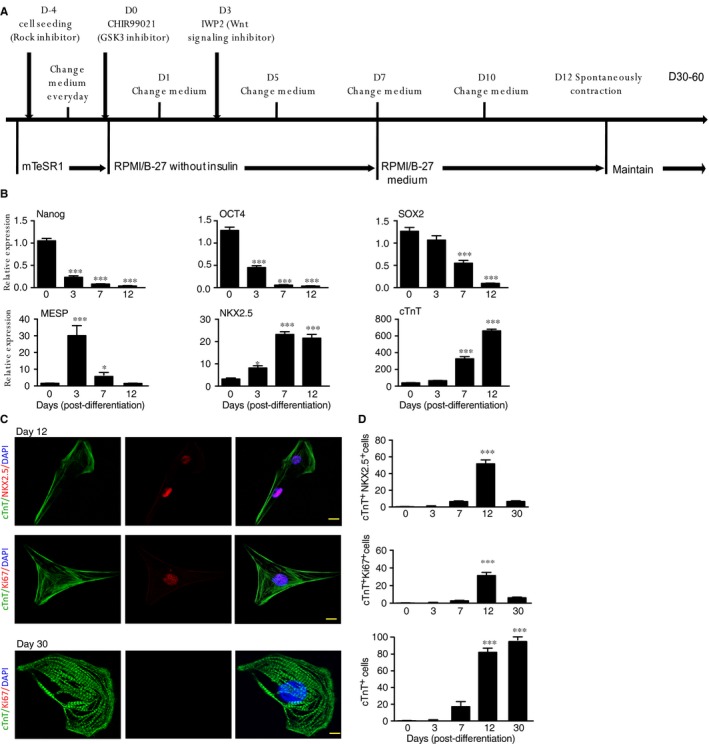
Characterization of cardiac lineage cells differentiated from hiPSCs. A, A protocol for in vitro differentiation of hiPSCs into cardiac lineage cells in a Matrigel. B, Relative expression of stem cell markers (Nanog, OCT4 and SOX2), CSC markers (MESP1 and NKX2.5), and CM marker cTnT during differentiation, C, Representative immunostaining images for CSC and CMs on day 12. D, Quantifications of cTnT^+^NKX2.5^+^ (day 12), cTnT^+^Ki67^+^ (day 12), cTnT^+^ Ki67^‐^(day 30). Scale bar: 10 μm. *Plt;0.05; ***Plt;0.001

In vitro differentiation from hESC or hiPSC has provided a useful approach to define the gene function in cell specification. A matrix sandwich protocol with the GSK3 inhibitor and Wnt inhibitor (GiWi protocol) has produced high yield preparations of CSC from hESC or hiPSC^27^. We employed the differentiation protocol from hiPSC into CSC/CMs (Figure. 1A). hiPSCs, reprogrammed from human dermal fibroblasts, expressed Yamanaka factor OCT4, SOX2and KLF4 (Figure S1). At day 12 of differentiation, the cells showed hallmarks of CMs, including spontaneous contraction.

We first performed quantitative RT‐PCR to detect the sequential gene expression during CSC differentiation. Stem cell markers Nanog, OCT4 and SOX2 were drastically decreased on day 3 of differentiation. Subsequently, early CSC marker MESP1, CSC markers, GATA4 and NKX2.5 were increased during differentiation, peaking at day 3–7 and declining by day 12 post‐differentiation. Differentiated cells started to express mature CM marker cTnT at day 7‐12 post‐differentiation concomitant spontaneous beating (Figure 1B). We used immunofluorescence to detect the expression of cardiac‐specific proteins in differentiated CSC and CMs. At day 12 of differentiation, more than 80% CSC/CMs expressed the cardiac‐specific myofilament cTnT, and among these cells 50% expressed NKX2.5 and 30% cells expressed Ki67(Figure 1C**;** Figure S2 for low power images). The resulting CMs progressively matured over 30 days in culture based on myofilament expression pattern and mitotic activity when mature CMs fully expressed myofilament expression with diminished mitotic activity (Ki67 staining) (Figure 1C).

**Figure 2:**
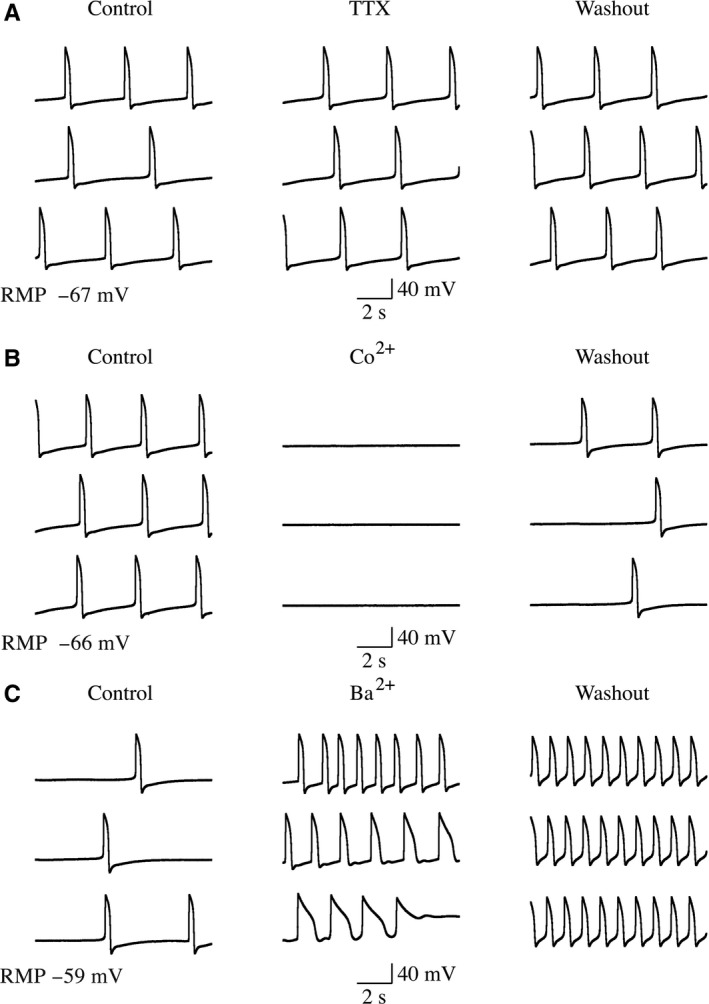
Functional maturity of differentiated CMs evaluated by electrophysiology. hiPSC‐based cardiac differentiation was performed and hiPSC‐derived CMs after day 30 differentiation were subjected to electrophysiology through single cell dissection from random areas and followed by action potential and calcium influx recordings in the whole cell patchclamp configuration. Representative traces of membrane potentials recorded from beating cells before, during and after the application of blockers of Na^+^ channel Tetrodotoxin (TTX, 1 μmol/L, A); Ca^2+^ channel (Co^2+^, 100 μmol/L, B); and K^+^ channel (Ba^2+^, 20 μmol/L, C)

Functional maturity of the differentiated CMs was evaluated by electrophysiology, which were determined through single cell dissection from random areas and followed by action potential and calcium influx recordings in the whole cell patchclamp configuration. A typical Ca^2+^(but not K^+^ or Na^+^) action potential was observed in hiPS‐derived CMs (Figure 2A–D). These data suggest that differentiated CMs not only express correct cellular markers but also exhibit functional properties of mature CMs.

### 3.2 TNFR2 expression precedes the expression of CSC markers in an in vitro differentiation system

**Figure 3:**
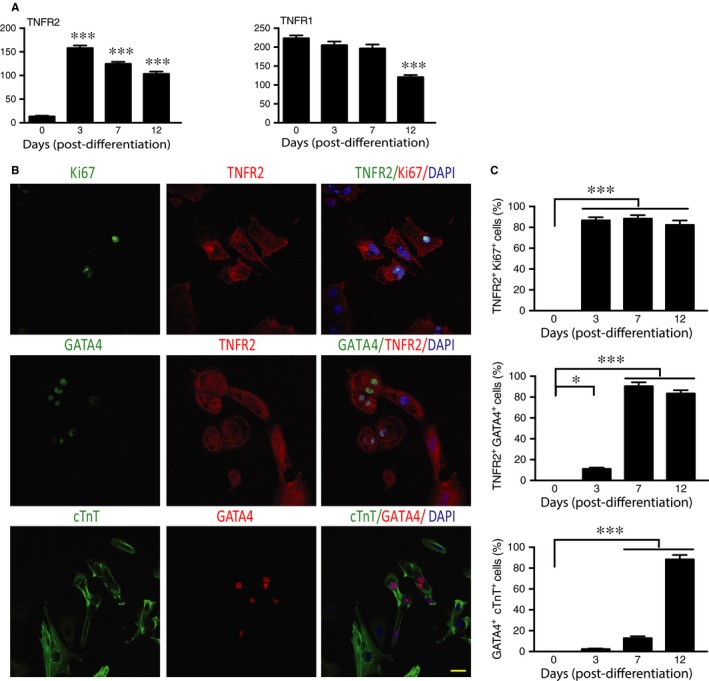
TNFR2 expression precedes cardiogenic markers during in vitro differentiation from hiPSCs. hiPSC‐based cardiac differentiation was performed. A, Relative expression of TNF receptors during differentiation B, Representative immunostaining images of TNFR2^+^ cells during differentiation. C, Quantifications of TNFR2^+^Ki67^+^, TNFR2^+^GATA4^+^ and GATA4^+^cTnT^+^ cells. Scale bar: 20 μm. *Plt;0.05; ***Plt;0.001

We examined gene expression of TNFR2 during differentiation and found that TNFR2 was highly up‐regulated upon differentiation but peaked at day 3 followed by a decline thereafter. In contrast, TNFR1 was ubiquitously expressed in all stages (Figure 3A). We evaluated expression of TNFR2 proteins and CSC markers by immunostaining. TNFR2^+^ cells could co‐express proliferative marker Ki67, CSC markers GATA4 and NKX2.5 in the in vitro differentiation system. Based on total number and percentages of positive cells, TNFR2^+^cells peaked on day 3, prior to appearance of TNFR2^+^GATA4^+^ and TNFR2^+^NKX2.5^+^ cells during differentiation. A high percentage of TNFR2^+^ cells exhibited NKX2.5^+^GATA4^+^ with proliferative marker Ki67 on day 7 followed by a decline on day 12 of differentiation (Figure 3B and C). Taken together, the early kinetics of TNFR2 expression suggests that TNFR2 may play a role in CSC differentiation, proliferation and maturation.

### 3.3 Inhibition of TNFR2 attenuates whereas TNFR2‐specific agonist enhances cardiac cell activation/differentiation

**Figure 4:**
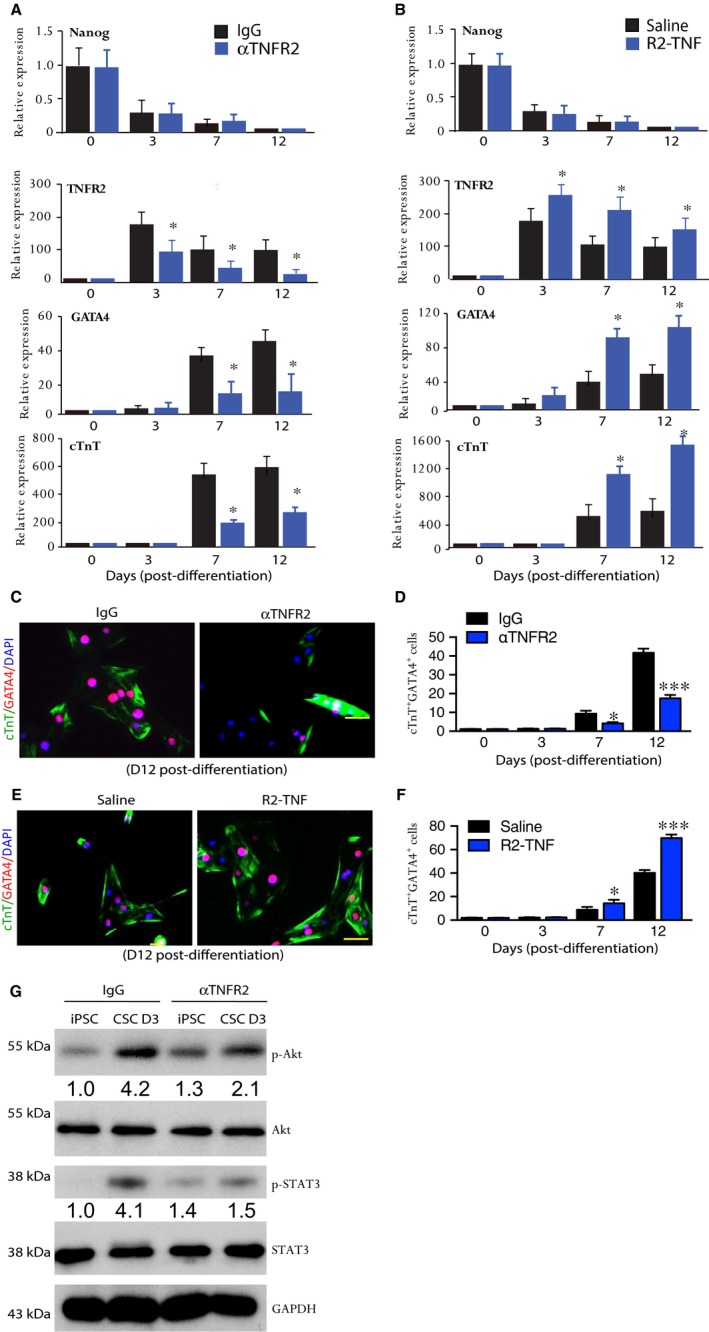
TNFR2 inhibition attenuates whereas TNFR2‐specific agonist enhances cardiac cell differentiation. hiPSC‐based cardiac differentiation was performed in the presence of isotype IgG or TNFR2 neutralization antibody (αTNFR2; 100 ng/ml) (A, C and D), or in the presence of Saline or R2‐TNF (100 ng/ml) (B, E and F). A and B, Relative expression of various markers during differentiation was determined by qRT‐PCR. Experiments were repeated three times. C to F, Representative immunostaining images of GATA4^+^cTnT^+^ cells are shown (C,E) and quantifications of GATA4^+^cTnT^+^ cells are presented (D,F). G, hiPSC‐based cardiac differentiation was performed in the presence of isotype IgG or TNFR2 neutralization antibody (αTNFR2; 100 ng/ml). hiPSC and D3 CSC lysates were subjected to Western blotting. Data are from three independent experiments. Scale bar: 50 μm. *Plt;0.05; ***Plt;0.001

We then tested our hypothesis that TNFR2 plays a critical role in CSC differentiation, proliferation and maturation. To this end, we examined the effect of TNFR2‐specific agonist (R2‐TNF) and TNFR2 neutralization antibody (αTNFR2) on CSC differentiation and maturation in the in vitro system. TNFR2‐specific ligand (R2‐TNF) with a site‐specific mutation (D143F) preferentially binds to TNFR2 and activates TNFR2‐specific signalling such as Akt (Figure S3). In contrast, TNFR2 neutralization antibody has been shown to block TNFR2‐dependent signalling^28^, ^29^. We observed that the presence of αTNFR2 or R2‐TNF in the differentiation media had no effect on gene expression of stem cell markers (such as OCT4). However, αTNFR2 drastically reduced, whereas R2‐TNF significantly increased, gene expression of CSC marker GATA4 and CM marker cTnT (Figure 4A and B). Accordingly, αTNFR2 attenuated while R2‐TNF augmented CSC differentiation and maturation as measured for GATA4 and cTnT immunostaining (Figure 4C–F).

To gain insight into the potential molecular mechanisms through which TNFR2 mediates CSC proliferation, differentiation and maturation, we examined the TNFR2 downstream signalling in CSC. We have previously reported that TNFR2 in vascular endothelial cells activates Akt and STAT3, leading to endothelial cell proliferation and migration^21^, ^40^, ^43^. These reports prompted us to determine if TNFR2 signalling induces Akt and STAT3 activation during CSC activation/differentiation. We detected both Akt and STAT3 were highly activated at early phase of CSC differentiation, coinciding with the kinetics of TNFR2 expression. Importantly, the presence of anti‐TNFR2 antibody (αTNFR2) blocked phosphorylation of Akt and STATA3 (4g), consistent with its effect on CSC activation/differentiation. These data suggest that TNFR2‐mediated Akt and STAT3 signalling is required for CSC proliferation, differentiation and maturation.

### 3.4 TNFR2 is up‐regulated by Lin28 at an early phase of CSC activation/differentiation

**Figure 5:**
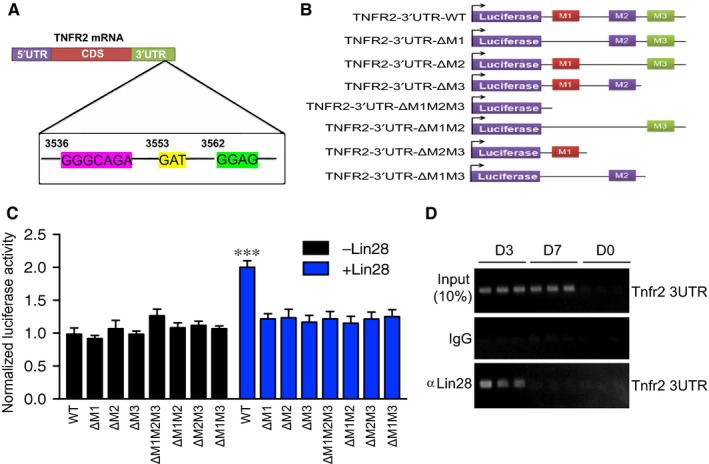
Lin28 regulates TNFR2 expression by directly binding to the Tnfr2mRNA 3’‐UTR. A, A diagram for the Tnfr2 mRNA 3’‐UTR containing a putative Lin28‐binding motif. B, Luciferase reporter gene constructs with WT or a mutant Tnfr2 mRNA 3’‐UTR. C, A Tnfr2 mRNA 3’‐UTR reporter gene plasmid was co‐transfected with a renilla reporter in the presence or absence of Lin28 expression plasmid into H9C2 cardiomyoblast cells. Relative luciferase activities are presented by normalization with renilla activity. *p < 0.05. D, Binding of Lin28 to the Tnfr2 mRNA 3’‐UTR at early phase (day 3) during cardiac differentiation as detected by RNA‐immunoprecipitation assay. Day 0 (iPSC), day 3 and day 7 post‐differentiation cells were used for the assays. An isotype IgG control was used as a control for anti‐Lin28. *Plt;0.05; ***Plt;0.001

Distinct from TNFR1,TNFR2 expression is restricted in certain cell types^30^. Expression of TNFR2 at an early stage of differentiation prior to CSC generation promoted us to examine if stem cell/pluripotent factors could regulate TNFR2 expression. Lin28 is an RNA‐binding protein that regulates microRNA generation and stability. It also regulates protein translation by binding to the 3’‐untranslated region (3'UTR) on mRNAs^31^. It has been reported that three conserved sequences ‘GGGCAGA’, ‘GAT’ and ‘GGAG’ on mRNA 3’‐UTR are within the consensus recognition motif for Lin28^32^. Sequence analyses indicated that the Tnfr2 mRNA 3’‐UTR contains such a motif (Figure 5A). The 3'UTR of Tnfr2 was inserted into a luciferase reporter plasmid (Luc‐Tnfr2‐3'UTR) followed by mutations at one or all three of the Lin28‐binding sequences (Tnfr2‐3'UTR‐ΔM1, ΔM2, ΔM3 and ΔM123) (Figure 5B). To determine if Lin28 enhances TNFR2 translation via the Tnfr2 3'UTR, an effect of Lin28 co‐expression on the Luc‐Tnfr2‐3'UTR reporter gene activity was analysed. Co‐expression of Lin28 increased activity of the Luc‐Tnfr2‐3'UTR reporter gene in H9C2 cardiomyoblast cells. However, a deletion at any one of three conserved sites diminished the effect of Lin28 on the reporter gene (Figure 5C). We further assessed the ability of Lin28 binds to the Tnfr2 3’‐UTR during CSC differentiation by an RNA‐binding protein immunoprecipitation (RIP) assay. Consistent with the kinetics of Lin28 and TNFR2 expression, the binding of Lin28 to the 3’‐UTR of Tnfr2 mRNA was not detectable in hiPSC at day 0, but was strongly detected in cells at day 3 of differentiation when Lin28^+^TNFR2^+^ cells peaked followed by a decline in day 7 when TNFR2^+^GATA4^+^ cells peaked (Figure 5D). Taken together, these results indicate that Lin28 up‐regulates TNFR2 expression at an early phase of CSC differentiation by transiently binding to the Tnfr2 3’‐UTR.

### 3.5 Inhibition Lin28 attenuates TNFR2 expression and cardiac cell activation/differentiation

**Figure 6:**
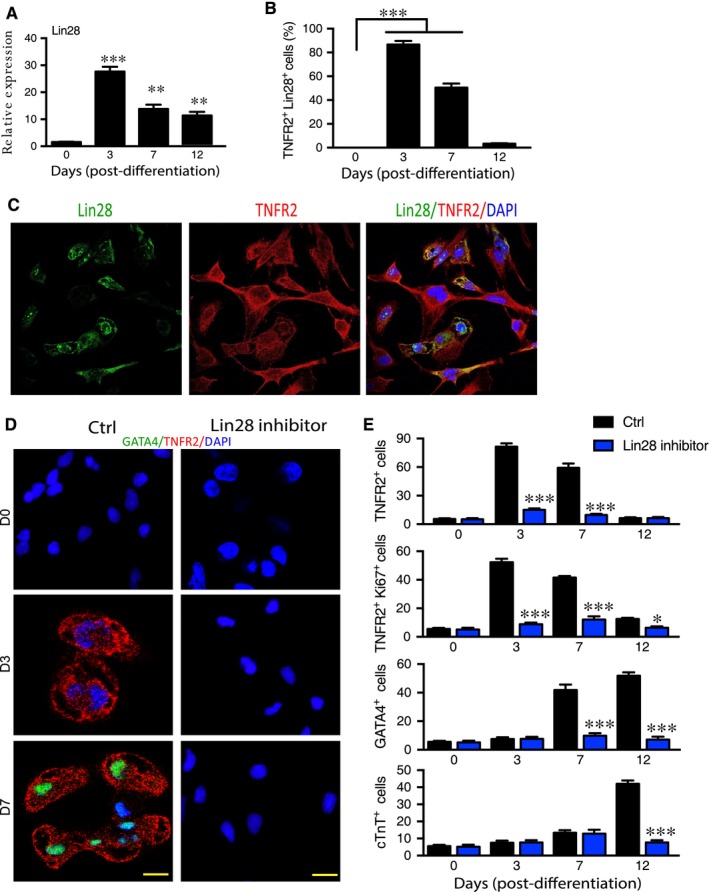
Lin28 regulates TNFR2 expression and cardiac differentiation. A‐C. hiPSC‐based cardiac differentiation was performed. Relative expression of Lin28 during differentiation. A, Quantifications of Lin28^+^TNFR2^+^ cells. B, Representative immunostaining images of Lin28^+^TNFR2^+^ cells on day 3. C, Scale bar: 10 μm. D and E. hiPSC‐based cardiac differentiation was performed in the absence or presence of Lin28 1632 (50 μmol/L). D, Representative immunostaining images of TNFR2^+^ and GATA4^+^ cells. E, Quantifications of TNFR2^+^, TNFR2^+^Ki67^+^ GATA4^+^ and cTnT^+^ cells. Scale bar: 20 μm. **Plt;0.01

We examined gene expression of Lin28 and TNFR2 during differentiation and found that Lin28, like TNFR2 was highly up‐regulated upon differentiation but peaked at day 3 followed by a decline thereafter (Figure 6A). TNFR2^+^ cells could co‐express Lin28 and Lin28^+^TNFR2^+^cells peaked on day 3, prior to appearance of TNFR2^+^GATA4^+^ and TNFR2^+^NKX2.5^+^ cells during differentiation (Figure 6B and C).

We then determined the role of Lin28‐mediated TNFR2 expression in hiPSC‐derived CSC differentiation. To this end, we examined effects of Lin28 inhibition on CSC differentiation. hiPSC‐based CSC differentiation was performed in the absence or presence of a Lin28 inhibitor Lin28 1632. Inhibition of Lin28 significantly reduced the number of total TNFR2^+^ cells and proliferating TNFR2^+^ cells as well as differentiated GATA4^+^ and cTnT^+^ cells as measured by immunostaining (Figure 6D and E).

## 4 DISCUSSION

**Figure 7:**
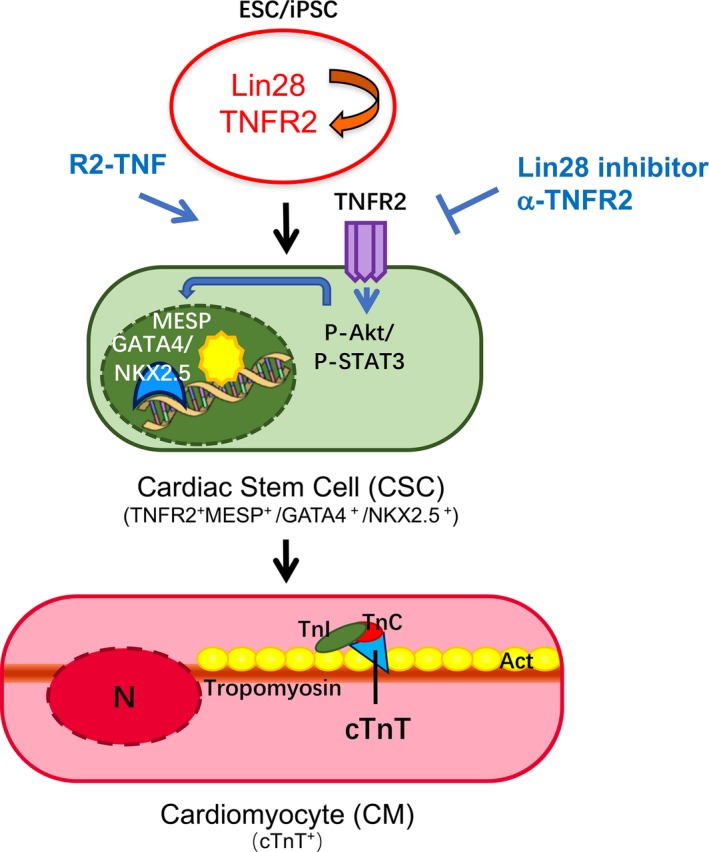
A model for the role of Lin28‐TNFR2 signalling in cardiac stem cell activation and differentiation. Lin28 induces TNFR2 expression in iPSCs. Proliferative TNFR2^+^ cells in turn become CSCs which subsequently become cTnT^+^ mature cardiomyocytes. TNFR2 may mediate Akt and STAT3 signalling to induce CSC activation and differentiation. TNFR2 inhibition attenuates whereas TNFR2‐specific agonist enhances cardiac cell activation/differentiation. CSC: cardiac stem cells; CM: cardiomyocytes; cTnT: cardiac troponin T; αR2: TNFR2 neutralization antibody; R2‐TNF: TNFR2‐specific agonist

TNFR2 has been implicated to have cardiac‐protective functions. Ablation of the TNFR2 gene exacerbates heart failure and reduces survival, whereas ablation of TNFR1 blunts TNF‐induced heart failure and improves survival in TNF‐transgenic mice^33^, ^34^. We have reported that TNFR1 and TNFR2 are differentially expressed in human ischaemic myocardium and proposed a cardioprotective role of TNFR2 in ischaemic heart^29^. Subsequently, we have shown that TNFR2^+^ cells with phospho‐histone H3^S10^ (pH3^S10^) are detected in human ischaemic heart and co‐express pluripotent stem cell protein Lin28^26^. However, it is unknown if and how TNFR2 signalling is required for CSC differentiation and how TNFR2 is regulated and activated during CSC differentiation. In this report, we have taken an in vitro approach of differentiation from hiPSC to CSC and we have found that TNFR2 expression is induced at an early phase of CSC differentiation. Specifically, Lin28 up‐regulates TNFR2 protein expression by directly binding to a conserved Lin28‐motif within the 3'UTR of Tnfr2 mRNA. Further kinetics analyses indicate that Lin28‐TNFR2 expression not only precedes the expression of CSC markers and mature CM proteins, but also is required for CSC generation. This is supported by the result that inhibition of Lin28 orTNFR2 diminishes, whereas TNFR2 activation by a specific agonist promotes, hiPSC‐based CSC differentiation, proliferation and maturation (Figure 7: Model for the role of Lin28‐TNFR2 signalling in CSC/CM activation and differentiation). A recent study suggests that TNF via TNFR1 inhibits cardiomyogenic commitment but promotes smooth muscle and endothelial fates during CSC differentiation. However, both TNFR1 and TNFR2 channel an alternate CSC neuroadrenergic‐like fate^35^. It would be interesting to determine if activation of TNFR2 alone by a specific agonist promotes CSC generation by suppressing the fate of smooth muscle cells, endothelial cells and neuroadrenergic‐like fate.

One important mechanistic finding in our study is that TNFR2 is up‐regulated in cardiogenic cells. It is known that TNFR2 expression is restricted to specific cell types such as endothelial cells and CMs , and can be induced under various pathological conditions, primarily at a transcriptional level. TNFR2 promoter contains several consensus elements for transcriptional factors SP1, AP1 and NF‐κB; all of these factors could be activated by inflammatory cytokines. Therefore, TNFR2 expression has been shown be regulated by cytokines, including interleukin‐1β and TNF itself^21^, ^30^, ^36^. Since TNFR2 is co‐expressed with MESP1, Lin28 as well as cardiogenic factors GATA4 and NKX2.5, we have reasoned that cardiogenic cells exhibit unique ability to turn on TNFR2 expression. Indeed, TNFR2 is up‐regulated in the in vitro hiPSC differentiation system. We further demonstrate that the pluripotent factor Lin28, an RNA‐binding protein, could directly bind to a consensus Lin28‐motif within the 3'UTR of Tnfr2 mRNA to up‐regulate TNFR2 protein expression. Lin28 is best known to regulate generation of miRNA let‐7, but also acts in let‐7‐independent fashion by either promoting or suppressing protein translations^37^, ^38^. Our data suggest that Lin28 promotes the TNFR2 translation by binding to its 3'UTR. Interestingly enough, it has been shown that Lin28 transcription can be strongly induced by inflammation‐activated NF‐κB and Lin28 in turn further enhance the NF‐κB‐dependent inflammatory responses, forming a positive feedback loop^32^, ^39^. It is plausible that inflammation activates Lin28 to induce TNFR2 expression in ischaemic heart. It needs to be determined how Lin28 is up‐regulated in the in vitro hiPSC differentiation system in the absence of inflammatory cytokines. Our data show that blockade TNFR2 reduces whereas R2‐TNF sustains Lin28 expression in the in vitro system, suggesting TNFR2 by activating NF‐κB could form feedback loop with Lin28. Of note, TNFR2‐specific activation promotes cell survival without enhancing inflammation as we have previously demonstrated in TNFR2‐transgenic mice^40^. Therefore, R2‐TNF together with hESC/hiPSC‐derived CSCs would provide an effective treatment for ischaemic heart disease.

A remaining question is the molecular mechanisms through which TNFR2 mediates CSC proliferation, differentiation and maturation. Recent studies suggest that both Akt and STAT3 are critical for CSC proliferation and differentiation from ESCs^41^, ^42^. We observe that both Akt and STAT3 are highly activated at early phase of CSC differentiation. Consistent with the effects of TNFR2 neutralization antibody on CSC activation/differentiation, anti‐TNFR2 antibody blocks activation of Akt and STATA3 during CSC differentiation. Previously we have identified Bmx, a non‐receptor tyrosine kinase implicated in cell migration, as the first TNFR2‐specific tyrosine kinase. TNFR1, via an adaptor molecule ASK1‐interacting protein‐1 (AIP1), activates ASK1‐JNK‐dependent cell apoptosis. In contrast, TNFR2 via Bmx promotes cell activation, migration, growth or proliferation in vascular endothelial cells^21^, ^40^, ^43^. Furthermore, we show that Bmx binds to the C‐terminal 16 aa sequence of TNFR2 to mediate TNFR2‐induced Akt and STATA3‐dependent cell migration and angiogenesis^21^, ^40^, ^44^. Importantly, both TNFR2 and Bmx have been implicated to have cardiac‐protective functions^29^, ^43^, ^45^, ^46^, ^47^, ^48^. It needs further investigations to determine if Bmx mediates TNFR2‐dependent Akt/STATA3 activation during CSC activation/differentiation.

Collectively, we have defined the important function of TNFR2 in CSCs activation and differentiation. Therefore, specific activation of TNFR2 signalling may be a novel strategy for the treatment of ischaemic diseases in humans.

## AUTHOR CONTRIBUTIONS

The following people designed, performed research and analysed data: QX, BY, LL,BQ, CQ, XBG, HJZ and WM; HJZ and WM wrote the paper.

## CONFLICT OF INTEREST

The authors confirm that there are no conflicts of interest.

## Data Availability

All other data supporting the presented findings are available from the corresponding author upon request.
