# Supplementary material for: Critical role of Lin28‐TNFR2 signalling in cardiac stem cell activation and differentiation
Source: J Cell Mol Med. 2019 Feb 7;23(4):2943–53. doi: 10.1111/jcmm.14202 (PMC6433861; doi:10.1111/jcmm.14202)
Supplement: Supplementary file 1 [file JCMM-23-2943-s001.pdf]

SUPPLEMENTARY DATA

Critical role of Lin28-TNFR2 signaling in cardiac stem cell activation and differentiation

Qiuling Xiang et al

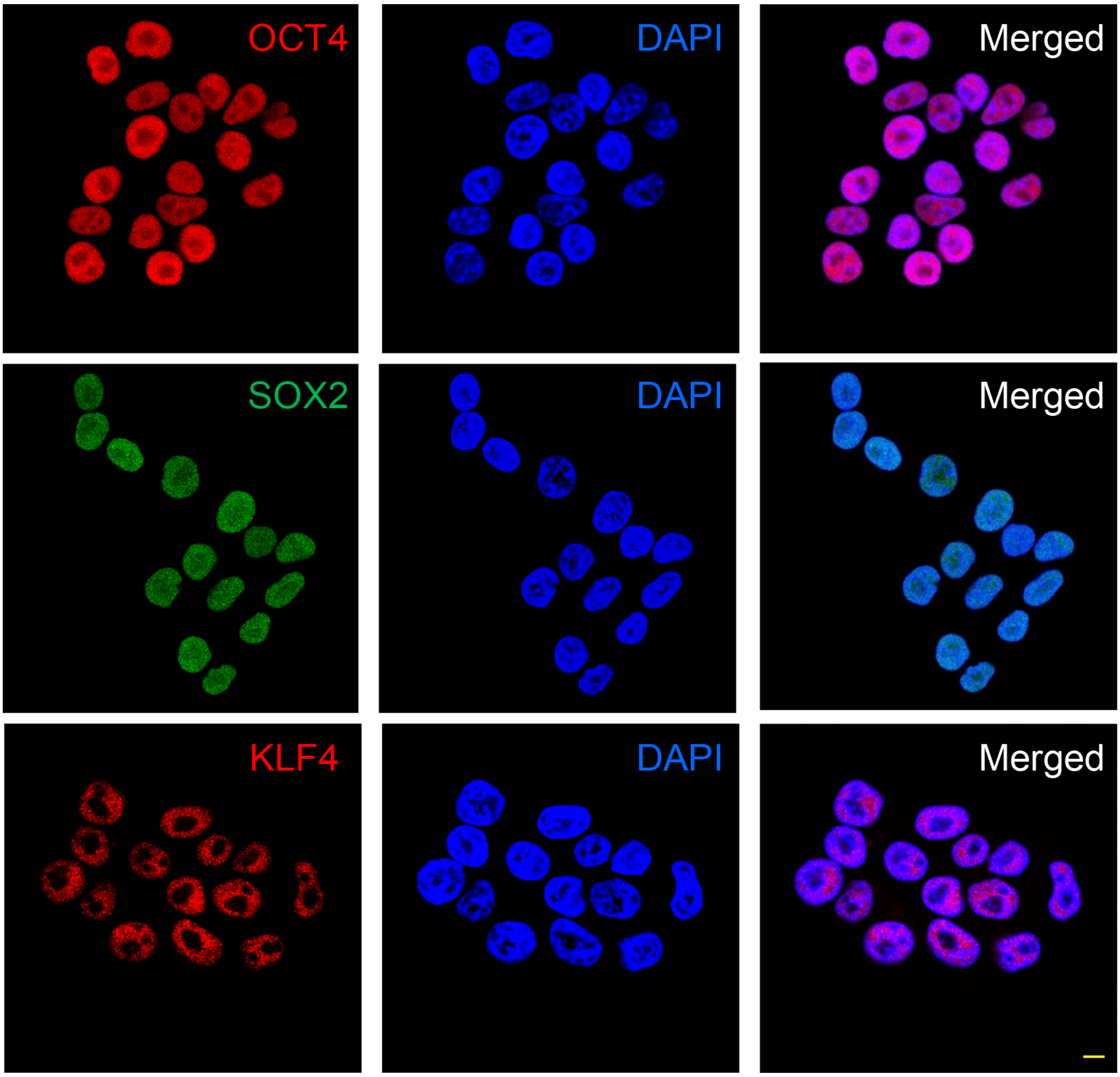

Supplementary Fig.S1. Characterization of hiPSCs. Cultured ihPSCs were subjected to immunofluorescence staining with stem markers. Representative immunostaining images for OCT4, SOX2 and KLF4. Scale bar: 10 μm.

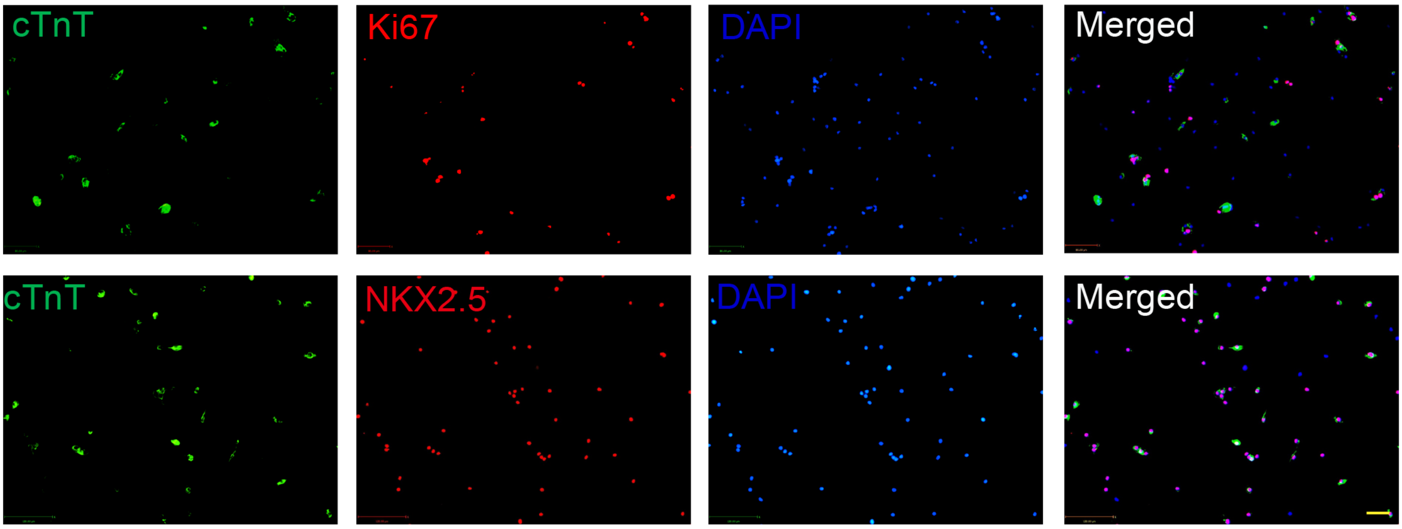

Supplementary Fig.S2. Characterization of hiPSCs-derived CSC. hiPSC-based cardiac differentiation was performed. Representative immunostaining images of $cTnT^{+}Ki67^{+}$ and $cTnT^{+}NKX2.5^{+}$ cells. Scale bar: 100 μm.

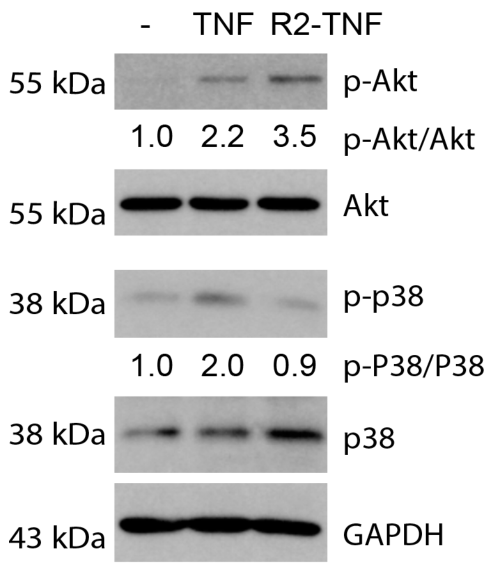

Supplementary Fig.S3. R2-TNF specifically induces activation of Akt but not p38 MAPK signaling. hiPSCs derived cardiogenic cells were treated with TNF-alpha (10 ng/ml) or R2-TNF as indicated (100 ng/ml) for 15 min. Cell lysates were subjected to Western blotting as indicated. Experiments were repeated three times.

Supplementary Table I Sequences of primers used in qPCR

Gene	F	R
hNANOG	TTTGTGGGCCTGAAGAAAACT	AGGGCTGTCCTGAATAAGCAG
hOCT4	GTGGAGGAAGCTGACAACAA	ATTCTCCAGGTTGCCTCTCA
hSOX2	GCCGAGTGGAAACTTTTGTCG	GGCAGCGTGTACTTATCCTTCT
hGATA4	CGACACCCCAATCTCGATATG	GTTGCACAGATAGTGACCCGT
hNKX2.5	CCAAGGACCCTAGAGCCGAA	ATAGGCGGGGTAGGCGTTAT
hTnnt2	TTCGACCTGCAGGAGAAGTT	GAAGGAGGCCAGGCTCTATT
hLin28	CCCAGTGGATGTCTTTGTGC	CAGACCCTTGGCTGACTTCT
hTNFR2	TGAAACATCAGACGTGGTGTG	TGCAAATATCCGTGGATGAAGTC
hTNFR1	TCTATGCCCGAGTCTCAACC	GGTGAGGGACCAGTCCAATA
18srRNA	CAGCCACCCGAGATTGAGCA	TAGTAGCGACGGGCGGTGTG

Supplementary Table II Antibodies used for immunostaining or immunoblotting

Antibody	Product number	Working Concentration	Application	Source
TNFR2	AB-226-PB(R&D)	1:300	IF	goat
Nkx2.5	SC-14033(Santa Cruz)	1:400	IF	rabbit
GATA4	SC-25310(Santa Cruz)	1:300	IF	mouse
cTnT	MA5-12960(ThermoFisher)	1:300	IF	mouse
Ki67	9129 (Cell Signaling)	1:400	IF	rabbit
Lin28	Ab46020(Abcam)	1:300	IF	rabbit
pH3s10	Ab5176(Abcam)	1:300	IF	rabbit
DAPI	Vector(H-1200)		IF	
p-Akt	4060s(Cell Signaling)	1:1000	IB	rabbit
Akt	4691(Cell Signaling)	1:1000	IB	rabbit
p-p38MAPK	4511s(Cell Signaling)	1:1000	IB	rabbit
p38MAPK	9212(Cell Signaling)	1:1000	IB	rabbit
p-STAT3 (Y705)	9131 (Cell Signaling)	1:1000	IB	rabbit
STAT3	9132 (cell Signaling)	1:1000	IB	rabbit
anti-rabbit IgG	7074s(Cell Signaling)	1:1000	IB	
anti-mouse IgG	7076(Cell Signaling)	1:1000	IB	
anti-goat IgG	SC-2354(Santa Cruz)	1:1000	IB	
GAPDH	2118s(Cell Signaling)	1:1000	IB	rabbit
